# Challenges to the implementation of telemedicine in abortion care for victims of sexual violence in Brazil

**DOI:** 10.3389/fgwh.2022.902390

**Published:** 2022-12-07

**Authors:** Beatriz Galli, Jina Dillon

**Affiliations:** ^1^ Senior Policy and Advocacy Consultant Ipas, Chapel Hill, NC, United States; ^2^ Technical Excellence Director Ipas, Chapel Hill, NC, United States

**Keywords:** abortion, Brazil, telemedicine, sexual violence, human rights, COVID-19 pandemic

## Abstract

The article focuses the recent dynamics resulting from state institutions adding more legal and regulatory barriers to abortion care access, particularly against the use of telemedicine for sexual violence victims in Brazil. It presents a case study from a lawsuit targeting a pioneer public health service on the city of Uberlandia to ban telemedicine in abortion care. The case study highlights human rights violations of women's right to health as well as the recent threats to the right to safe legal abortion care. It also provides legal arguments—based on scientific evidence and international human rights standards—that support the use of telemedicine for abortion care.

## Introduction

In Brazil, abortion is a crime under the Penal Code but allowed in two circumstances: (i) if there is no other way to save the life of the pregnant woman and (ii) if the pregnancy results from rape (Article 128, II, Penal Code) (1). In 2012, another circumstance was considered legal for pregnancy termination, following a decision of the Federal Supreme Court, which is (iii) when the woman is pregnant with an anencephalic fetus (2). The Technical Guideline from the Ministry of Health on Abortion Humanized Care establishes as requirements for access to legal abortion in public health services: the consent of a woman over 18 years of age, and the participation of a legal representative, as assistant or representative, of the child and adolescent (3).

The Covid-19 pandemic has offered an opportunity to implement telemedicine abortion care to sexual violence victims in Brazil based on a new legal framework. A new law was enacted to expand the allowance of the use of telemedicine (4). Additionally, the Ministry of Health has included telehealth consultation in primary care as a procedure of the Unified Health System (SUS), free and universal (5).

Moreover, new regulations of the Brazilian sanitary surveillance agency (ANVISA) were enacted allowing the delivery and dispensing of medicines under special control to be delivered at home if the other access criteria were fulfilled (6). This legal change was an opportunity to expand access to misoprostol during the pandemic through telemedicine abortion care. As Prandini and Erdman have argued, misoprostol has a double life in Brazil as an essential medicine and controlled drug (7) (p4). It is included both in the list of essential medicine for obstetric care use and is included in the list of medicines under special control by ANVISA.

Due to the Covid-19 pandemic, a deficit was found in the provision and availability of legal abortion service to sexual violence victims due to closures of outpatient clinics (8). Some states were not offering a single referral center, and in others, most services were available only in capitals, mainly in the Southeast region (9). Although any hospital with an obstetric practice should be able to perform legal abortions, during the Covid-19 pandemic it was observed that the number of hospitals offering legal abortion procedures dropped (10).

In Brazil due to the restrictive legislation on abortion, black women and adolescents who live in poverty, in rural and other isolated areas or who are victims of domestic and sexual violence, lack the information, means, and ability to make autonomous decisions about their sexuality and life plans. This reality was exacerbated by the Covid-19 pandemic (11).

New regulations were enacted by the government during the pandemic adding more barriers to already limited circumstances in which abortion is legally allowed in Brazil. The Ministry of Health published a new regulation establishing mandatory reporting from health providers to the police in cases of sexual violence. This regulation was the Ordinance of the Ministry of Health No. 2,282 from August 27th, 2020 (12) which also included a provision by which providers should offer a health exam with fetus image to pregnant women after rape. Later, after strong mobilization from public institutions and civil society organizations, it was replaced by Ordinance 2.561 from September 23rd, 2020, which removed the referred provision but maintained the duty of mandatory police reporting by providers in the Procedure for Justification and Authorization of Interruption of Pregnancy, within the scope of the Unified Health System-SUS (13).

The Penal Code from 1940 does not establish a gestational limit or requires police reporting or judicial authorization for access to abortion in cases of rape. Mandatory reporting of rape victims without their consent is illegal, and violates the human right to health, and the rights to confidentiality and privacy in health care as protected in the Brazilian Constitution and international human rights treaties ratified by the Brazilian state, such as the Convention on the Elimination of All Forms of Discrimination against Women (14).

## Methodology considerations

The methodology chosen to present the Brazilian scenario on the challenges to the use of telemedicine for sexual violence victims is the case study. Case studies can be used to *explain, describe,* or *explore* events or phenomena in the contexts in which they occur (15). The case study approach is useful to capturing information on more explanatory “*how*”, “*what”* and “*why*” questions, such as “*how* is the intervention being implemented and received on the ground?”. It can offer additional insights into *what* gaps exist in its delivery or *why* one implementation strategy might be chosen over another (16).

This paper focuses on a particular situation in the Brazilian unique context: the political controverse around the implementation of a comprehensive reproductive health program, in particular telemedicine for abortion care for sexual violence victims. The Brazil case study presented describes advocacy strategies and the legal arguments in context to promote the safety and effectiveness of the use of telemedicine for abortion care, with the aim of influencing courts' decisions in this area based in international human rights law and global human rights standards adopted by the 2022 WHO Abortion Care Guideline (17).

## Brazil case study

In United Sates, telemedicine for abortion care was considered safe, cost-effective, and the preferred method of abortion during acute periods of COVID-19 transmission (18). A study found examples of eight countries where governments removed regulatory barriers to the practice of telemedicine abortion in response to the pandemic (19). In the United Kingdom, on March 30, 2020, the Department of Health and Welfare liberalized the regulation of legal abortion for two years, or while the Coronavirus Law is in effect, allowing legal abortion service by telemedicine as a temporary measure broadening its scope for the pregnant person to receive medicines by mail and for home use (20).

Similarly, in France, early abortion *via* telemedicine was allowed in response to the difficulties in accessing the service in the pandemic. The Minister of Health's Decree of April 14, 2020, approves the use of telemedicine and abortion with medicines at home until nine weeks of pregnancy, also allowing the drug to be purchased in pharmacy (21).

Telemedicine provides an opportunity to expand access to abortion care in restrictive settings, as proven in the Brazilian scenario during the Covid-19 pandemic. Telemedicine is a model of health service delivery where providers and clients are separated by distance. It can improve the availability, accessibility, and acceptability of health care for people who experience barriers due to poverty, distance from a health care facility, or discrimination (19). It is recommended by the World Health Organization as an alternative to in-person interactions for provision of medical abortion services in whole or in part (17). According to the data available, self-administration of the drug can be as successful and effective among women in abortion care as provider administration in the hospital (22).

The implementation of telemedicine for abortion care for sexual violence victims was an important step to improve effectiveness and availability of legal abortion services in Brazil (23). This initiative was firstly implemented at the Comprehensive Care Center for Victims of Sexual Assault, also called NUAVIDAS, located at Hospital from the Federal University of Uberlândia, state of Minas Gerais.

In partnership with the feminist organization Anis Institute, NUAVIDAS health staff developed the Protocol for Legal Abortion *via* Telehealth in which self-management of misoprostol was allowed at home for pregnancy termination, with remote supervision by health staff outside from the health facility (24). The protocol adopted by NUAVIDAS followed international human rights standards and best scientific-based evidence available on telemedicine in abortion care, by which pregnant women exercise the right to informed consent, autonomy in decision making and right to privacy in abortion care (25).

Despite positive results and documented health outcomes, in July 2021, a public civil lawsuit was presented against the Ministry of Health administration asking for the immediate suspension of NUAVIDAS program using telemedicine in abortion care, requesting to the Court to declare its illegality “in the entire national territory, of any medical services provided by booklets or protocols that promote the procedure of legal abortion remotely, without follow-up in person physician and with the use of the drug misoprostol outside the hospital environment” (26). The action was dismissed without judgment on the merits. Many civil society organizations have been presenting amicus briefs on behalf of NUAVIDAS evidence-based telemedicine abortion care based on constitutional rights and international human rights law.

In June 2022 the Ministry of Health released the Guide “Technical Attention for Prevention, Assessment and Conduct in Abortion Cases” (the Guide) containing a series of misconceptions and illegalities not based on scientific evidence (27). The Guide also erroneously affirms that unsafe abortion is not among the leading causes of maternal mortality and that the numbers of unsafe abortion are inflated for ideological reasons (28). It does not adopt a human rights-based approach to every preventable death and ignores the fact that causes of maternal deaths and injuries are underreported when restrictive laws are in place (17). It further states that “every abortion is a crime, but when situations of exclusion of illegality are proven after police investigation, it is no longer punished, as termination of pregnancy due to maternal risk.” (29).

Legal abortion in Brazil is allowed by law for sexual violence victims and a police investigation is not required for its performance in public health services. The Guide if applied can potentially add delays in service provision and promote fear of investigation to victims of violence that seek health care since police reporting can happen without their consent, thereby violating their human rights to autonomy, privacy, and confidentiality in health care. The Penal Code of 1940 in its Article 154 establishes as a crime of violation of professional secrecy “revealing someone, without just cause, a secret, of which they are aware by reason of their function, ministry, trade or profession, and whose disclosure may cause harm to others (30).

The Guide states that abortion *via* telehealth is illegal, and therefore not authorized” (31) conflicting with current legislation on telemedicine, best scientific evidence, health, and human rights standards established by WHO guidelines. The denial of access to telemedicine abortion care after rape to sexual violence victims leads to intersectional discrimination on more vulnerable women and girls living in rural and poor urban areas distant from public health facilities and without economic means of transportation, in their majority poor, black or indigenous (32).

The Guide also erroneously refers to an absolute protection of life under Brazilian Constitution (33) contradicting the 2011 Ministry of Health Technical Guideline on Humanized Care for Abortion, which is still in force. This regulation includes as a requirement for access to legal abortion merely victms' informed consent (3), adopting a human rights-based principles to abortion care.

In June 2022, as a reaction to the recent regulatory changes with additional and unnecessary barriers to sexual violence victims' access to abortion care, Brazilian civil society organizations presented a constitutional remedy called Action for Breach of Fundamental Precept before the Federal Supreme Court. They argued that the state should be held accountable due to the enactment of additional regulatory barriers aggravating quality provision of legal abortion in cases of sexual violence and requesting specific measures to address violations to fundamental rights in Brazil (34).

## Discussion

During the pandemic, gaps and barriers affecting availability of abortion care services for sexual violence victims were exacerbated in Brazil. Telemedicine for abortion care was firstly implemented in a referral public health facility for sexual violence victims in the city of Uberlandia. Their protocol expanded legal interpretation to implement telemedicine abortion care to sexual violence victims under remote supervision from hospital health staff and in accordance with international human right standards and evidence-based care.

Misoprostol has a double standard in Brazil. It is a lifesaving and an essential drug included in the List of Essential Drugs in Brazil since 2010, but it is included in the list of medication under special control under ANVISA regulations limiting its accessibility and availability to sexual violence victims in need (35). The purchase of misoprostol in pharmacies is not legally allowed in Brazil due to very restrictive regulations by which the drug label is only for “hospital use” (36).

NUAVIDAS protocol included hospital-supplied misoprostol with supervised use, allowing women to self-administer the drug in their households with telemedicine support, in the pandemic (37). The Federal Public Prosecutor's Office in Uberlandia supported and declared the legality of NUAVIDAS model of telemedicine for abortion care to sexual violence victims (26).

Data indicate that with the use of misoprostol to terminate pregnancy in countries with restrictive laws, the number of abortion complications has dropped considerably, despite the difficulties in access (38). The WHO classification for unsafe abortion has adopted a category of less safe – less unsafe-considering this change in the global landscape following the widespread use of misoprostol by women in restrictive legal contexts (39).

The human right to health comprises the right to the benefits of scientific progress, including the human right to have access to an essential and lifesaving drug, based on equality and non-discrimination in health care. Abortion regulations reducing barriers to access abortion pills that allow use of misoprostol outside health facilities without prescription or its direct purchase in pharmacies are based on science and in line with international human right standards (40).

Brazil is a state party to key international human rights treaties such as the Convention on the Rights of the Child (1989); the Convention on the Elimination of All Forms of Racial Discrimination (1966); the Convention on the Elimination of All Forms of Discrimination against Women (1979); and the International Pact on Economic, Social and Cultural Rights (1966), among others. However, the Brazilian state has yet to take measures to respect, protect and fulfill human rights for sexual violence victims, bringing national laws and policies in line with its international human rights obligations (41).

At the national level, the right to health is constitutionally guaranteed, under the terms of Article 6 of the Brazilian Constitution, as a social right (42). In addition, the Article 196, “health is the right of all and the duty of the State, granted by means of social and economic policies that aim at reducing the risk of disease and of other maladies, and at providing universal and equal access to the actions and services that promote health, protection and recovery” (43).

Governments have the obligation under human rights law to repeal or eliminate laws, policies and practices that criminalize, obstruct, or undermine an individual's or a particular group's access to health facilities, services, goods, and information, including abortion (44). The Brazilian state violates human rights standards when it establishes mandatory reporting to the police in cases of rape, prohibits access to telemedicine abortion care and restricts access to an essential medicine such as misoprostol to sexual violence victims.

The Committee on the Elimination of Violence against Women (CEDAW), for example, recommended to states to provide all health services in a manner consistent with women's human rights, including the rights to autonomy, privacy, confidentiality, informed consent, and choice (45) [CEDAW GR 24, paragraph 31(e)]. In addition, the Committee on Economic Social and Cultural Rights (CESCR) on General Comment 22, on the Right to Sexual and Reproductive Health under Article 12 of the International Covenant on Economic Social and Cultural Rights, calls for the repeal or reform of discriminatory laws, policies and practices in the area of sexual and reproductive health, including liberalization of restrictive abortion laws, as well as the removal of all barriers that interfere with access by women to comprehensive sexual and reproductive health services, goods, education and information (46). (CESCR, GC 22 pars. 1–2).

## Conclusion

The political and legal environment for abortion care access to sexual violence victims has deteriorated during the pandemic with less services available and restrictive regulations in place with mandatory police reporting from health staff. In June 2022, the Ministry of Health issued a new Guide prohibiting access to telemedicine abortion care. The Guide ignores human rights standards and science-based evidence from the World Health Organization, imposing more legal and policy barriers to an already very restrictive environment.

Brazil is a case example of systematic human rights violations by state neglect, omission, and commission, particularly denial of safe abortion care to sexual violence victims. An expected human right and evidence-based response from the Brazilian state during the pandemic would be to ease access to legal abortion care through telemedicine to every victim of sexual violence in need. Denying access to abortion care in these circumstances is a form of gender-based violence, according to international human rights standards developed by UN human rights bodies (47).

The pioneer model of telemedicine for abortion care called NUAVIDAS, was the first public health service to use telemedicine for legal abortion care in the country. This initiative has been studied and documented, with positive results impacting women and adolescents’ health and rights (48). However, the NUAVIDAS telemedicine for abortion care model to sexual violence victims has been challenged in court.

By adding barriers to health care and prohibiting the use of telemedicine for sexual violence victims, the Brazilian government denies essential services, discriminates women, girls and pregnant people who were raped and are in desperate need of abortion care violating their human rights. Current efforts by several civil society organizations to respond to this scenario have been articulated through strategic litigation to secure constitutional rights and access to legal abortion care. This is an example of long-term advocacy strategy in defense of sexual reproductive health and rights particularly the right to safe abortion for sexual violence victims in Brazil.
